# Importance of Beta Cell Function for the Treatment of Type 2 Diabetes

**DOI:** 10.3390/jcm3030923

**Published:** 2014-08-14

**Authors:** Yoshifumi Saisho

**Affiliations:** Department of Internal Medicine, Keio University School of Medicine, 35 Shinanomachi, Shinjuku-ku, Tokyo 160-8582, Japan; E-Mail: ysaisho@z5.keio.jp; Tel.: +81-3-3353-1211 (ext. 62383); Fax: +81-3-3359-2745

**Keywords:** type 2 diabetes, beta cell, insulin secretion, therapy

## Abstract

Type 2 diabetes (T2DM) is characterized by insulin resistance and beta cell dysfunction. Recent evidence has emerged that beta cell dysfunction is a common pathogenetic feature of both type 1 and type 2 diabetes, and T2DM never develops without beta cell dysfunction. Therefore, treatment of T2DM should aim to restore beta cell function. Although the treatment of T2DM has greatly improved over the past few decades, remaining issues in the current treatment of T2DM include (1) hypoglycemia; (2) body weight gain; (3) peripheral hyperinsulinemia and (4) postprandial hyperglycemia, which are all associated with inappropriate insulin supplementation, again underpinning the important role of endogenous and physiological insulin secretion in the management of T2DM. This review summarizes the current knowledge on beta cell function in T2DM and discusses the treatment strategy for T2DM in relation to beta cell dysfunction.

## 1. Introduction

The incidence of diabetes is increasing worldwide. According to the International Diabetes Federation (IDF), there were estimated to be 382 million patients with diabetes in 2013, which is expected to increase to 592 million by 2035 [1]. Diabetes caused 5.1 million deaths, and health spending on diabetes accounted for 10.8% of total health expenditure worldwide in 2013 [1]. In Japan, diabetes causes ~4000 cases of blindness, and ~17,000 patients with diabetes commence hemodialysis annually. Of patients with diabetes, type 2 diabetes (T2DM) comprises 90%–95%; however, currently no effective strategy to prevent or cure T2DM is available.

T2DM is characterized by insulin resistance and beta cell dysfunction [2]. However, recent evidence has emerged that beta cell dysfunction is a core pathogenetic mechanism of diabetes and T2DM develops only when beta cell function is impaired [3]. This review summarizes the current knowledge on beta cell dysfunction in T2DM and discusses the treatment strategy for T2DM in relation to beta cell function.

## 2. Deficit of Beta Cell Function and Mass in T2DM

T2DM is characterized by insulin resistance and beta cell dysfunction [2]. Since plasma insulin level is often higher in patients with T2DM compared with non-diabetic individuals, T2DM is characterized by obesity, hyperinsulinemia, and insulin resistance; however, the significance of beta cell dysfunction in patients with T2DM has been often ignored.

However, recent studies have consistently shown the presence of beta cell dysfunction in patients with T2DM [4]. Since insulin secretion shows a compensatory increase in the presence of insulin resistance, true beta cell function should be evaluated based on insulin secretion adjusted by insulin sensitivity, the so-called disposition index [5]. Using the disposition index, DeFronzo *et al.* have reported that beta cell function is already decreased by approximately 80% in patients with impaired glucose tolerance (IGT) compared with non-diabetic subjects [4].

Moreover, recent studies have shown that not only beta cell function, but also beta cell mass is decreased in patients with T2DM. Butler *et al.* conducted histological analysis of beta cell mass in the pancreas obtained from autopsy subjects with or without T2DM [6]. As a result, they found that beta cell mass was decreased by approximately 40% and 65% in lean and obese individuals with T2DM, respectively, compared to age- and BMI-matched non-diabetic individuals. Other histological studies have also confirmed reduced beta cell mass in patients with T2DM [7,8,9].

Taken together, the current evidence consistently shows that there is reduced functional mass of beta cells in patients with T2DM. Since type 1 diabetes (T1DM) is characterized by beta cell loss due to autoimmune attack [10], the current evidence indicates that reduced beta cell mass is a common pathophysiological feature of both type 1 and type 2 diabetes.

## 3. Beta Cell Function and Glycemic Control

If reduced functional beta cell mass is a common pathophysiological feature of both type 1 and type 2 diabetes, what is the clinical relevance of beta cell dysfunction in T2DM? Since beta cell function is already reduced in patients with IGT, preceding the onset of T2DM, beta cell dysfunction has an important role in the deterioration of glucose tolerance [4]. Thus, preservation or recovery of beta cell function could be an effective therapeutic strategy to prevent T2DM [11].

**Figure 1:**
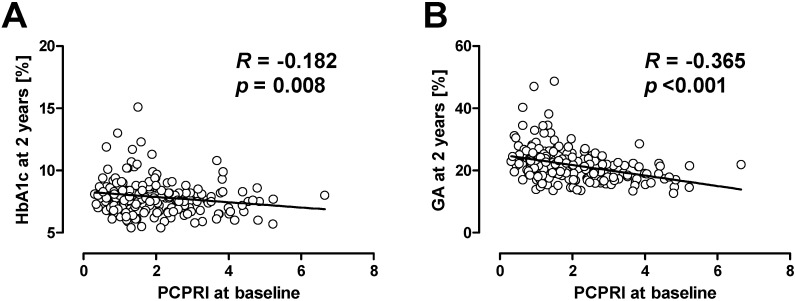
Correlation between baseline postprandial C-peptide index (PCPRI) and HbA1c (**A**) and glycated albumin (GA) (**B**) after two years. Reproduced with permission from [20].

Recent studies also suggest the importance of beta cell function in the management of hyperglycemia in patients with T2DM. In the UK Prospective Diabetes Study (UKPDS) [12] and a Diabetes Outcome Progression Trial (ADOPT) [13], treatment failure was associated with a progressive decline of beta cell function [14,15]. The association between beta cell dysfunction and treatment failure is not only observed in adult patients, but was also shown in adolescent patients with T2DM. The Treatment Options for type 2 Diabetes in Adolescents and Youth (TODAY) trial showed that lower beta cell function at baseline was associated with poorer glycemic control after four years in adolescent patients with T2DM who were treated with metformin or metformin plus rosiglitazone [16]. In our retrospective cohort study, we also evaluated the association between beta cell function and clinical outcome in Japanese patients with T2DM [17,18]. Beta cell function was evaluated by serum or urinary C-peptide level. As a result, we found that patients with lower beta cell function at baseline were more likely to subsequently need insulin therapy compared with those with higher beta cell function. It is of note that among C-peptide indices, postprandial C-peptide index (*i.e.*, 2 h postprandial serum C-peptide (ng/mL)/plasma glucose (mg/dL) × 100) was the best predictor of subsequent need for insulin therapy compared with fasting C-peptide index and urinary C-peptide [17,19]. More surprisingly, even with more frequent insulin therapy, patients with lower beta cell function at baseline showed higher glycated hemoglobin (HbA1c) and glycated albumin (GA) levels after 2 years (Figure 1) [18,20]. These results indicate that beta cell function is significantly associated with clinical outcome, *i.e.*, glycemic control, in patients with T2DM.

**Figure 2:**
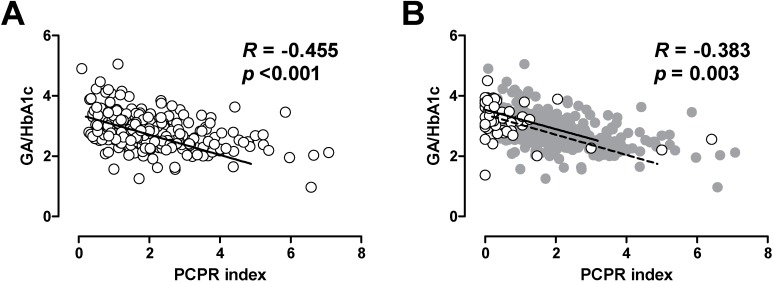
Correlation between postprandial C-peptide index (PCPRI) and glycated albumin (GA) to HbA1c ratio in patients with type 2 diabetes (**A**) and type 1 diabetes (**B**). In Figure 2B, the data of patients with type 1 diabetes are superimposed on the data of those with type 2 diabetes (gray circles and dotted line). Reproduced with permission from [24].

In patients with T1DM, it has been shown that lower residual C-peptide is associated with greater glycemic fluctuation [21,22,23]. Thus, we also evaluated the association between postprandial C-peptide index and glycemic excursion in patients with T2DM [24]. Since glycated albumin (GA) is more rapidly glycated than is HbA1c, GA or GA to HbA1c ratio more sensitively reflects postprandial glycemic excursions compared with HbA1c [24,25,26]. We, and others, have reported that postprandial C-peptide index is negatively correlated with GA to HbA1c ratio in patients with T2DM [24,27], indicating that lower beta cell function is associated with greater postprandial glycemic excursions in patients with T2DM. Interestingly, the relationship between postprandial C-peptide index and GA to HbA1c ratio was comparable between patients with T1DM and T2DM (Figure 2) [24,25], indicating that beta cell function similarly affects glycemic fluctuation in patients with T1DM and T2DM. These results further underpin the importance of beta cell function in both types of diabetes.

Thus, recent studies have shown that lower beta cell function is associated with a higher rate of treatment failure, poorer glycemic control and greater glycemic fluctuation in patients with T2DM, which are probably related to higher risk of diabetic complications in these patients, as suggested by several studies [28,29].

## 4. Current Issues in Treatment of Type 2 Diabetes: Inappropriate Insulin Supplementation

Current issues in the treatment of T2DM are summarized in Table 1.

**Table 1:** Current issues in treatment of type 2 diabetes.

Issue	Cause
Hypoglycemia	Excess insulin
Weight gain	Excess insulin
Concern of increased risk of malignancy and/or atherosclerosis	Excess insulin, especially peripheral hyperinsulinemia
Postprandial hyperglycemia	Insufficient insulin in postprandial state, especially in portal vein

### 4.1. Hypoglycemia

T2DM is associated with a two- to four-fold increased risk of cardiovascular disease (CVD) [30]. As shown in the UKPDS, intensive glycemic control in patients with T2DM effectively suppressed the development or progression of diabetic microangiopathy; *i.e.*, retinopathy, nephropathy and neuropathy, during the study; however, the effect of intensive glycemic control on CVD was less apparent [31], although a significant reduction in the rate of CVD was obtained after 10 years of study [32]. Recent randomized controlled trials (RCTs) aiming to control HbA1c to near normal levels (*i.e.*, HbA1c < 6%–6.5%) failed to show a reduction of CVD [33,34,35], and in one trial, the Action to Control Cardiovascular Risk in Diabetes (ACCORD) trial, all-cause mortality was rather significantly increased in the intensive therapy group compared with the conventional therapy group. In the ACCORD trial, the intensive therapy group showed a higher incidence of hypoglycemia including severe hypoglycemia and greater weight gain compared with the conventional therapy group during the study [34].

Hypoglycemia is the most frequent and serious adverse event associated with the treatment of diabetes. Intensive glycemic control is inversely correlated with increased risk of hypoglycemia [31,33,34,35]. Hypoglycemia not only reduces patients’ quality of life (QOL) but also appears to induce inflammation, blood coagulation abnormality, sympathoadrenal response, and endothelial dysfunction, which could affect the onset of CVD [36,37], suggesting that hypoglycemia increases the risk of CVD.

### 4.2. Weight Gain

Weight gain associated with treatment of T2DM is also a major issue. Use of sulfonylureas (SU) and thiazolidinediones (TZD) is usually associated with weight gain [38]. Insulin therapy also often induces weight gain. Use of metformin is associated with less weight gain or even weight loss compared with SU or TZD treatment [13,39,40,41,42,43]. The effect of dipeptidyl peptidase-4 (DPP-4) inhibitors on body weight has been shown to be neutral, while glucagon-like peptide-1 (GLP-1) receptor agonists (GLP-1RA) have a favorable effect on body weight [44,45]. Weight gain not only may result in treatment failure but also may induce other obesity-related diseases and impair QOL [46].

### 4.3. Peripheral Hyperinsulinemia

The third issue is the concern that diabetes treatment may increase the risk of cancer and/or atherosclerosis. In 2009, a study reported by Hemkens *et al.* showed a significant increase in overall cancer risk by use of insulin glargine compared with human insulin [47]. However, papers reported by other groups did not find such a correlation [48,49,50]. A number of subsequent analyses including a subanalysis of randomized controlled trials also have not found any significant association between insulin glargine and risk of cancer [51,52,53,54], resulting in no warning label or restriction on the use of insulin glargine with regard to cancer to date. Nonetheless, the study by Hemkens *et al.* indeed showed a similar increase in risk of cancer or all-cause mortality between insulin glargine and human insulin in their Cox model adjusted for age and sex [47]. More recently, Currie *et al.* have examined the correlation between glycemic control and all-cause mortality [55]. They found a general U-shaped curve between HbA1c level and all-cause mortality. This relationship was observed in both patients treated with oral hypoglycemic agents (SU and/or metformin) and those treated with insulin, with the lowest hazard ratio (HR) at HbA1c of around 7.5%. HR for all-cause mortality was significantly higher in people given insulin-based regimens *vs.* those given combination oral agents (1.49, 95% confidence interval (CI) 1.39–1.59). Although the higher mortality in insulin-treated patients might simply reflect the severity of the disease, these findings indicate that the relationship between insulin therapy and mortality remains controversial. When insulin is subcutaneously injected, the distribution of insulin is not physiological, and systemic, rather than portal, hyperinsulinemia occurs. Although it has been reported that insulin has anti-inflammatory and anti-oxidative effects [56], insulin also has the potential to induce cell growth [57]. Thus, it has been suggested that peripheral hyperinsulinemia caused by subcutaneous insulin injection may promote tumor growth [57]. Peripheral hyperinsulinemia may also induce proliferation of vascular endothelial cells and increase plaque vulnerability within atherosclerotic plaques [58], which may increase the risk of CVD in insulin-treated patients [59].

The Outcome Reduction with Initial Glargine Intervention (ORIGIN) trial was designed to examine the effect of early basal insulin supplementation on the development of CVD in patients with IGT or T2DM [60]. A total of 12,537 patients participated, and the study showed that the effect of basal insulin supplementation was neutral in terms of development of CVD. In this study, no increase in cancer incidence in patients treated with insulin was also noted.

Although there is no evidence showing a causal relationship between insulin therapy and cancer or atherosclerosis to date [57,61], more physiological delivery of insulin to reproduce normal endogenous insulin secretion seen in people with normal glucose tolerance is clearly warranted.

### 4.4. Postprandial Glycemic Excursion

Hyperglycemia consists of two components; fasting hyperglycemia and postprandial glycemic excursion. Epidemiological studies have shown that postprandial hyperglycemia rather than fasting hyperglycemia is more strongly correlated with CVD and all-cause mortality [62,63,64]. It has also been shown that intermittent exposure to high glucose more strongly induces apoptosis in endothelial cells compared with chronic high glucose exposure in an *in vitro* study [65]. The recent development of continuous glucose monitoring systems (CGMS) has allowed more precise evaluation of the daily glycemic profile. Using this system, Monnier *et al.* [66] have reported a strong positive association between the mean amplitude of glycemic excursions (MAGE), a measure of daily glycemic variability, and urinary 8-*iso*-prostaglandin (PG) F2alpha excretion rate in patients with T2DM, indicating that greater glycemic variability results in greater production of oxidative stress in patients with T2DM, which may cause vascular damage and diabetic complications. Thus, reducing postprandial glycemic excursion and glycemic variability in patients with T2DM may improve CVD outcomes, although the role of glycemic variability in CVD outcome remains controversial [67,68,69,70].

**Figure 3:**
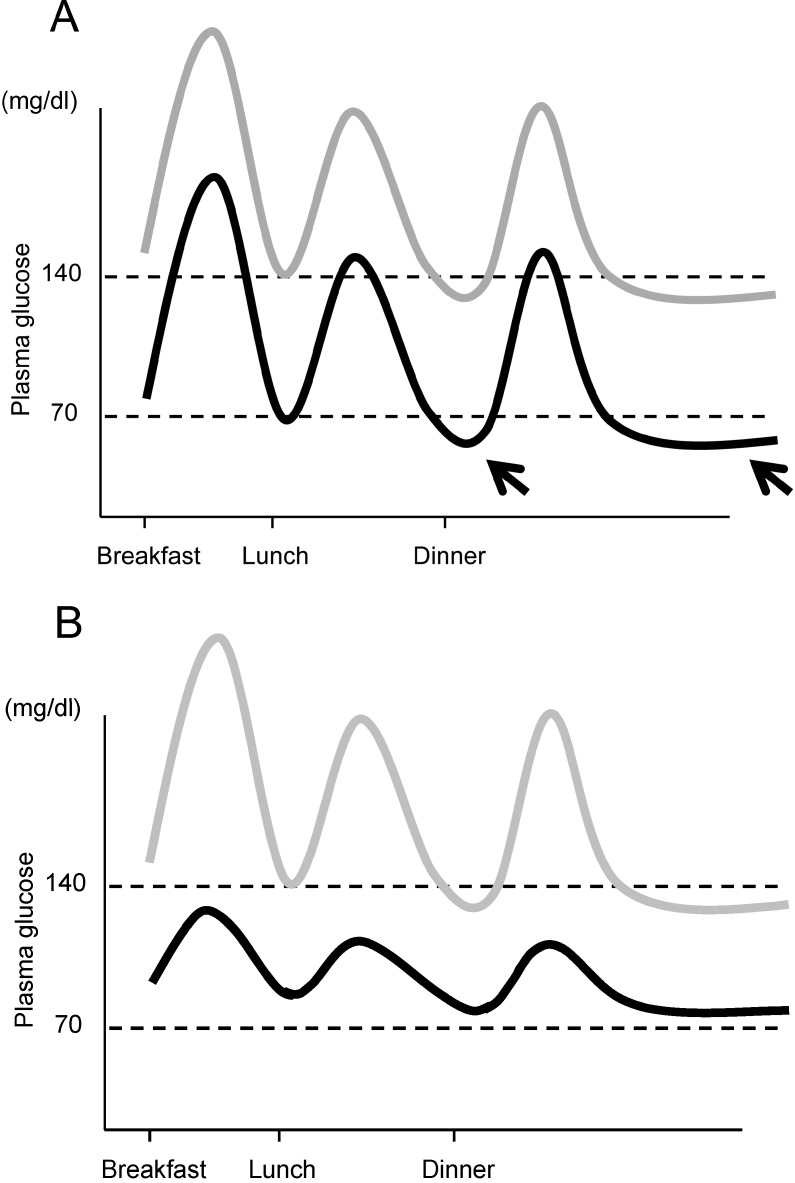
Importance of controlling postprandial glycemic excursion. (**A**) If mean plasma glucose level is lowered without controlling postprandial glycemic excursion (gray line → black line), the risk of hypoglycemia between meals and during the night increases (arrows); (**B**) Lowering the mean glucose level with control of postprandial glycemic excursion (gray line → black line) results in a low risk of hypoglycemia. Note that mean plasma glucose levels are similar in both cases, indicating similar HbA1c in both cases.

Moreover, another important aspect of managing postprandial glycemic excursion and glycemic variability is the prevention of hypoglycemia. The importance of reducing glycemic variability for prevention of hypoglycemia is illustrated in Figure 3. HbA1c reflects the average glucose. When lowering HbA1c, if glycemic variability persists, the risk of hypoglycemia increases (Figure 3A). We and others have reported that postprandial glycemic excursion and glycemic variability are increased in older patients with T2DM [25,71], and a greater risk of hypoglycemia has been reported in elderly patients with T2DM [72]. Thus, controlling postprandial glucose excursion and reducing glycemic variability are necessary to achieve good glycemic control without an increase in incidence of hypoglycemia, especially in the elderly.

## 5. Therapeutic Strategy in Relation to Beta Cell Function

The current issues in the treatment of T2DM described above are, simply said, all related to beta cell function; *i.e.*, excess or insufficiency of insulin supply (Table 1). Therefore, to overcome these issues of current therapy of T2DM, it is inevitably important to preserve or recover endogenous beta cell function and physiological insulin secretion in patients with T2DM.

To date, the most effective therapeutic strategy to preserve or recover beta cell function is likely to be to reduce beta cell workload or induce beta cell rest. These include lifestyle modification and/or weight reduction, and use of metformin or TZD. Lifestyle modification, *i.e.*, nutritional therapy and increase in physical activity, and weight reduction improve insulin sensitivity and thereby reduce beta cell workload. Intensive lifestyle modification with more than 7% weight loss suppressed the progression to T2DM by ~58% in patients with IGT [73]. In the same study, metformin therapy also reduced the progression to T2DM by ~31% [73]. Metformin improves insulin sensitivity mainly through suppressing hepatic glucose production, which results in reduced beta cell workload. TZDs have also been shown to effectively suppress the progression from IGT to T2DM [74,75,76]. TZDs improve insulin sensitivity in adipose tissue, thereby reducing beta cell workload. Insulin therapy is initiated to reproduce the physiological daily insulin profile. Insulin therapy has been shown to improve beta cell function [77,78], probably through inducing beta cell rest. In the ORIGIN study, adding basal insulin has been shown to suppress progression from IGT to T2DM [60]. On the other hand, nateglinide, a short-acting insulin secretagogue, failed to show a reduction in progression to T2DM in patients with IGT [79], suggesting that the therapeutic strategy to increase beta cell workload may not be effective to prevent deterioration of glucose metabolism.

Drugs to reduce beta cell workload have also been shown to be more effective to maintain optimal glycemic control compared with drugs to increase beta cell workload in patients with recent-onset T2DM. In the ADOPT study, treatment with metformin or rosiglitazone monotherapy resulted in a lower rate of treatment failure compared with SU monotherapy [13].

**Figure 4:**
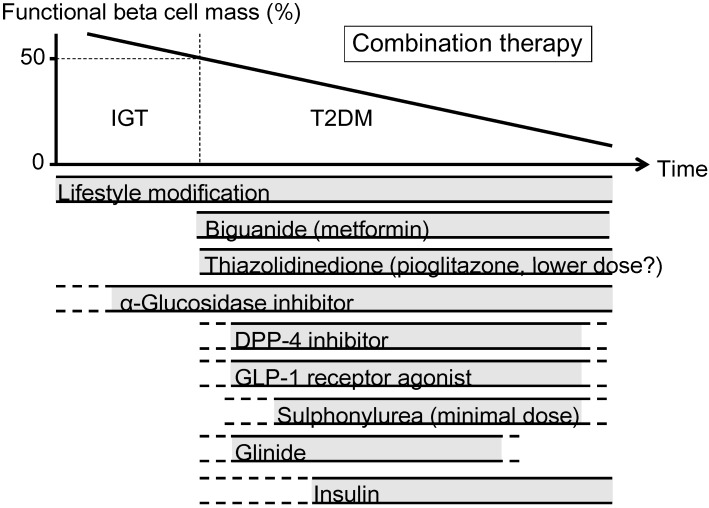
Proposed concept of treatment strategy for type 2 diabetes (T2DM) in relation to functional beta cell mass. An α-glucosidase inhibitor is partly approved for use in patients with impaired glucose tolerance (IGT) in Japan. Medications not approved or marketed in Japan are not included in the figure. Reproduced with permission from [101]. Since currently no single therapy or agent can cure and even manage T2DM, an effective combination of current medications in addition to lifestyle modification aiming at reduction in beta cell workload is important to preserve or recover beta cell function.

The currently proposed therapeutic strategy for T2DM aiming at preservation and/or recovery of beta cell function is shown in Figure 4. It is emphasized that, to reduce beta cell workload, lifestyle modification and weight reduction remain the most important therapy at any stage of T2DM. Although lifestyle modification failed to reduce the incidence of CVD in the Look AHEAD (Action for Health in Diabetes) trial [80], it has been reported that lifestyle modification improved cardiovascular risk factors, reduced the need for and cost of medication, reduced the rate of sleep apnea and urinary incontinence, improved well-being, and increased the rate of diabetes remission [81,82,83,84,85].

Metformin is positioned as first-line therapy in most guidelines for the treatment of T2DM [38,86]. Since metformin is effective in lean patients as well as obese patients with T2DM [87,88], it should be used in both lean and obese individuals unless contraindicated. Its efficacy in reducing HbA1c (~1.5%), low risk of hypoglycemia, favorable effect on body weight and low cost also support metformin as a first-line drug.

TZDs have been also shown to reduce beta cell workload and maintain glycemic control in the long term [13,15]. Rosiglitazone has been shown to increase low-density lipoprotein (LDL) cholesterol and the risk of coronary heart disease in patients with T2DM [89], and its use has been suspended or strictly restricted in Europe and USA [90,91], although recently the U.S. Food and Drug Administration (FDA) has lifted most of its restrictions [92]. On the other hand, pioglitazone has been shown to suppress the progression of atherosclerosis and reduce the risk of cardiovascular disease [93,94,95,96]. However, TZDs often induce weight gain and edema due to fluid retention, and are contraindicated in patients with heart failure [38]. Recent studies have also shown an increase in risk of bone fracture in women [97] and risk of bladder cancer [98,99,100] in patients treated with pioglitazone. The risk of bladder cancer may be dose dependent. In addition, since low-dose pioglitazone also reduces the risk of weight gain and edema, it may be preferable to use pioglitazone at lower doses, especially in women. Pioglitazone also should be used with caution in postmenopausal women with osteoporosis due to the increased fracture risk.

α-Glucosidase inhibitors (AGIs) delay the absorption of carbohydrate from the small intestine, and thereby reduce postprandial hyperglycemia, resulting in reduced beta cell workload in a postprandial state. AGIs have also been reported to reduce the progression to T2DM in patients with IGT [102,103]. Improving postprandial hyperglycemia by AGIs may also improve the cardiovascular outcome [104,105,106]. Therefore, although the reduction in HbA1c by AGIs is relatively small (~0.5%), their use is also considered in patients with T2DM, especially those with postprandial hyperglycemia. The major side effect of AGIs is gastrointestinal disturbance such as flatulence, diarrhea and abdominal pain. In Japan, AGIs are the only medication indicated for patients with IGT. Thus, AGIs are also considered for the treatment of T2DM at the early stage of the disease, if tolerated.

On the other hand, the use of insulin secretagogues, which increase beta cell workload, may be somewhat limited. SUs, while they remain among the most prescribed drugs for the treatment of T2DM, increase the risk of hypoglycemia and weight gain, resulting in a high rate of treatment failure [13]. These issues of SUs may be derived from their non-physiological augmentation of insulin secretion from beta cells.

Incretin drugs include DPP-4 inhibitors and GLP-1RAs. Both drug types reduce HbA1c mainly through an increase in insulin secretion, but also through suppression of glucagon secretion [107]. GLP-1RAs also slow gastric emptying and reduce appetite, resulting in weight loss. The most important characteristic of incretin drugs is probably that the enhancement of insulin secretion occurs in a glucose-dependent manner. Thus, the action of incretin drugs as insulin secretagogues is more physiological than that of SUs, thereby resulting in a low risk of hypoglycemia and weight gain with incretin therapy [44,108,109]. Whether this physiological enhancement of insulin secretion results in long-term maintenance of glycemic control remains to be elucidated. Although an increase in beta cell mass with incretin therapy has been reported in rodent studies [110,111], this effect has not been confirmed in humans [112,113,114]. As incretin therapy is usually well tolerated without serious adverse effects, the use of incretin drugs is rapidly increasing [115].

Glinides, short-acting insulin secretagogues, enhance early-phase insulin secretion, thereby reducing postprandial hyperglycemia [116]. As a defect in early-phase insulin secretion is a hallmark of glucose intolerance [117], enhancement of early-phase insulin secretion but not prolonged hyperinsulinemia by glinides is more physiological, unlike SUs, and is assumed to increase beta cell workload as well as risk of hypoglycemia to a lesser degree compared with SUs.

Thus, the use of insulin secretagogues may be limited because of an increase in beta cell workload as well as increased risk of hypoglycemia. As incretin enhances insulin secretion in a more physiological fashion, and is also expected to improve beta cell function and/or mass, incretin drugs could be used at any stage of T2DM. On the other hand, SUs may be used rather to enhance incretin action at only a minimal dose. To recover physiological insulin secretion, a combination of an incretin drug and a glinide may also be useful.

Insulin has been shown to improve beta cell function in patients with IGT and T2DM [60,77,78]. Since initial intensive insulin therapy has been shown to preserve beta cell function thereafter [76], insulin therapy should be considered as early as possible in patients with T2DM. Insulin therapy is also the most effective medication to reduce HbA1c [38]. However, increased risk of hypoglycemia, weight gain and non-physiological insulin delivery (*i.e.*, systemic *vs.* portal), in addition to fear of injections, limit its use. Insulin therapy to overpower insulin resistance without eliminating excess calories may worsen ectopic lipid overload [118].

A sodium-glucose cotransporter 2 (SGLT2) inhibitor has been recently approved in several countries including USA, EU, and Japan. SGLT2 inhibitors suppress reabsorption of glucose by SGLT2 in the proximal renal tubule and increase glucose excretion in urine (~60–80 g glucose/day) [119]. As a result, SGLT2 inhibitors not only decrease HbA1c, but also reduce body weight and blood pressure and improve the lipid profile. The action of SGLT2 inhibitors is independent of insulin. Thus, the efficacy of SGLT2 inhibitors seems to be regardless of beta cell function. SGLT2 inhibitors show a low risk of hypoglycemia but increase the incidence of bacterial urinary tract infections and fungal genital infections especially in women. Higher risk of hypotension has also been reported [120]. SGLT2 inhibitors may be suitable for obese patients with T2DM and metabolic syndrome; however, their longer-term safety including cardiovascular and cancer risk and efficacy remain unknown [120,121].

**Figure 5:**
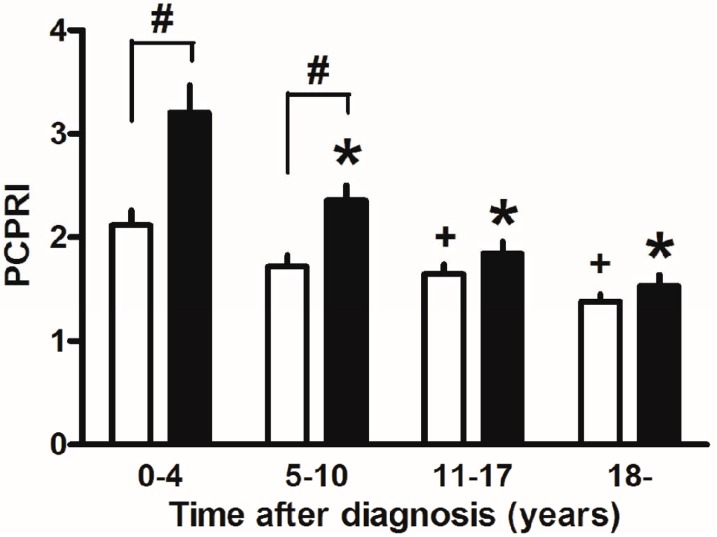
Postprandial C-peptide index (PCPRI) in subjects according to obesity and time after diagnosis (0–4, 5–10, 11–17 and ≥18 years). There were significant differences in PCPRI between lean (open bars) and obese subjects (solid bars) in the first and second quartiles of time after diagnosis, but no significant differences were observed in the third and fourth quartiles. * *p* < 0.05 *vs.* obese subjects ≤4 years after diagnosis, + *p* < 0.05 *vs.* lean subjects ≤4 years after diagnosis, # *p* < 0.05 *vs.* lean subjects. Reproduced with permission from [123].

Finally, marked weight reduction by bariatric surgery such as gastric bypass and sleeve gastrectomy has been reported to markedly improve glycemic control and even achieve remission of T2DM in severely obese T2DM patients [122]. This also suggests the importance of reducing beta cell workload, although change in incretin secretion has also been proposed as another mechanism by which glucose metabolism is improved after gastric bypass. In our retrospective cohort, the progressive decline in beta cell function seemed exaggerated in the presence of obesity in Japanese patients with T2DM (Figure 5) [123]. The remission of T2DM after bariatric surgery is associated with residual beta cell function [124,125], indicating the importance of residual beta cell function to manage and/or cure T2DM.

## 6. Conclusions

**Figure 6:**
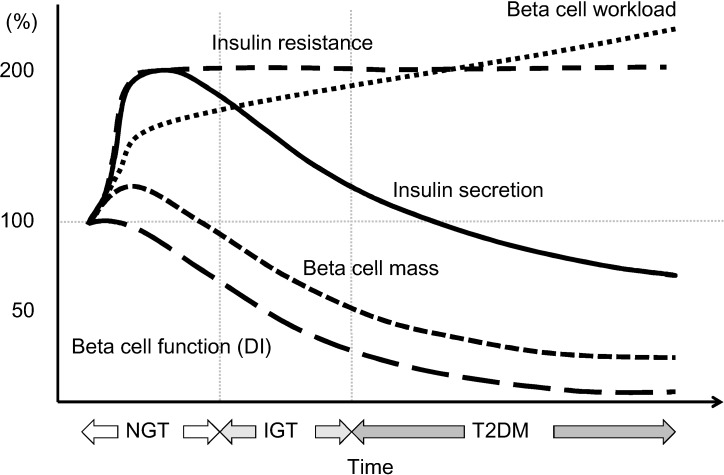
Hypothesis of change in beta cell function and mass during development of abnormal glucose tolerance. The magnitude of the increased demand for insulin due to insulin resistance caused by excess caloric intake and physical inactivity exceeds the magnitude of beta cell mass expansion, resulting in an increase in beta cell workload. In individuals who are susceptible to type 2 diabetes (T2DM), increased beta cell workload may lead to beta cell failure and the development of T2DM. Reproduced with permission from [101].

This review summarizes the current knowledge on beta cell dysfunction in T2DM and discusses the treatment strategy for T2DM in relation to beta cell function. The hypothetical change in beta cell function, beta cell mass and resulting beta cell workload is shown in Figure 6. Beta cell dysfunction resulting from increased beta cell workload due to genetic and/or environmental factors such as excess caloric intake and physical inactivity is central in the pathogenesis of T2DM, and beta cell function progressively declines with disease duration. Beta cell function relates to glycemic control, and thereby possibly relates to complications and mortality in patients with T2DM. Although a reduction in beta cell workload seems an effective therapeutic strategy to prevent or manage T2DM, currently, no single therapy or agent can cure and even manage T2DM. Thus, an effective combination of current medications in addition to lifestyle modification aiming at reduction in beta cell workload is important to preserve or recover beta cell function and achieve life-long optimal glycemic control in patients with T2DM. Furthermore, progressive decline in beta cell functional mass even prior to the development of diabetes emphasizes the importance of lifestyle modification for prevention of the disease. Such efforts may lead to a further reduction in the incidence of diabetic complications and increase longevity in patients with T2DM.
